# Selective estrogen receptor modulators decrease invasiveness in pituitary adenoma cell lines AtT‐20 and TtT/GF by affecting expression of MMP‐14 and ADAM12

**DOI:** 10.1002/2211-5463.12999

**Published:** 2020-10-26

**Authors:** Zhuo Zhang, Jörg W. Bartsch, Julia Benzel, Ting Lei, Christopher Nimsky, Benjamin Voellger

**Affiliations:** ^1^ Department of Neurosurgery University Hospital Marburg Germany; ^2^ Department of Neurosurgery Tongji Hospital Tongji Medical College of Huazhong University of Science and Technology Wuhan China; ^3^ Deutsches Krebsforschungszentrum (DKFZ) Heidelberg Germany

**Keywords:** ADAM proteases, anti‐estrogens, invasiveness, matrix metalloproteinases, pituitary adenoma

## Abstract

Selective estrogen receptor modulators (SERMs) significantly affect survival and invasiveness of rodent pituitary adenoma (PA) cells. The impact of three clinically relevant SERMs (bazedoxifene, clomiphene, raloxifene) on invasiveness and on gene and protein expression of invasion‐related proteases [matrix metalloproteinase‐14 (MMP‐14) and A disintegrin and metalloproteinase‐12 (ADAM12)] was analyzed in murine PA cells (AtT‐20 and TtT/GF). All SERMs significantly decreased cell invasiveness. Moreover, SERMs significantly decreased expression of ADAM12 mRNA in both cell lines and of MMP‐14 mRNA in TtT/GF cells. Invasion rates of AtT‐20 and TtT/GF significantly decreased after ADAM12 gene silencing, and the invasion rate of TtT/GF cells significantly decreased after MMP‐14 gene silencing. All SERMs affected ADAM12 protein expression in AtT‐20 cells whereas bazedoxifene and raloxifene decreased MMP‐14 protein expression in TtT/GF cells. We conclude that SERMs attenuate invasiveness of murine PA cells by downregulating expression levels of invasion‐related proteases MMP‐14 and ADAM12.

## Graphical abstract

Clinically, pituitary adenomas (PAs) become detrimental when invasive. We conducted an efficacy analysis of estrogen receptor modulators (SERMs) in PA cells. All SERMs blocked invasiveness of PA cells, correlating with expression levels of invasion‐related metalloproteases ADAM12 and MMP‐14. Our findings support the notion that SERMs are able to block invasiveness of PA cells by reducing their expression levels.

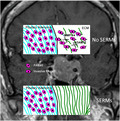

**Abbreviations**
ADAM: a disintegrin and metalloproteinase
ctrl, control; DAPI: 4′,6‐diamidino‐2‐phenylindole
GAPDH: glyceraldehyde 3‐phosphate dehydrogenase
IC: inhibitory concentration
min: minute
MMP: matrix metalloproteinase
PA: pituitary adenoma
qPCR: quantitative real time‐polymerase chain reaction
RIPA: radioimmunoprecipitation assay
rpm: revolutions per minute
SEM: standard error of the mean
SERM: selective estrogen receptor modulator
scr, scramble; siRNA: small interfering ribonucleic acid
TGF: transforming growth factor
TMZ: temozolomide

Pituitary adenomas (PAs) may exert a mass effect on neighboring tissues, and they may lead to endocrine dysfunction or cranial nerve deficiencies. Therapeutic options comprise surgery, radiation, and pharmacotherapy. The extent of surgical resection is often limited in invasive or recurrent PAs. Radiation therapy, which is usually applied with remnants or recurrence of PAs at an inoperable site, may affect neighboring tissues and induce tumors. Pharmacotherapy is useful only in certain PAs, depending on the hormones and receptors expressed in the tumor cells [1].

In 2011, McCormack *et al*. [2] published a careful analysis of reports on the potential usefulness of temozolomide (TMZ) in aggressive PAs. In 2018, the European Society of Endocrinology has recommended TMZ as first‐line chemotherapy in cases of aggressive PAs that are refractory to standard treatment [3]. However, a positive treatment effect of TMZ was only observed in 47 percent of cases [3]. Other than that, there is no widely recommended or evidence‐based chemotherapeutic option to treat aggressive PAs.

Several *in vitro* studies suggest antitumorigenic effects of selective estrogen receptor modulators (SERMs) like resveratrol [4, 5, 6] or clinically applicable drugs like bazedoxifene, clomiphene, and raloxifene [7] on rodent PA cells. In acromegalic men, the SERM raloxifene has been shown to reduce insulin‐like growth factor 1 levels [8]. There are indications that SERMs have an impact on cell‐extracellular matrix interaction which may subsequently affect invasiveness, for example, in breast cancer cells: Tamoxifen, a SERM, has been shown to reduce extracellular transforming growth factor‐beta 1 (TGF‐beta1) expression, while inhibition of matrix metalloproteinase (MMP) activity restored TGF‐beta1 levels in estrogen receptor‐positive breast cancer cells [9]. Reduced disease‐free survival time has been reported in patients with estrogen receptor‐negative and MMP‐9‐positive breast cancer [10]. Induction of MMP‐9 expression by laminin in a human cervical cancer cell line [11] and upregulation of MMP‐9 as well as coordinated expression of MMP‐2 and MMP‐14 by fibronectin in a human T lymphocyte cell line [12] have been demonstrated. MMP‐14 and a disintegrin and metalloproteinase‐12 (ADAM12) levels in human PA tissue samples are correlated with cavernous sinus invasion [13].

Here, we aim to investigate the impact of three clinically applicable SERMs, namely bazedoxifene, clomiphene, and raloxifene, on invasiveness *in vitro* and correlate their effects with gene and protein expression levels of MMP‐14 and ADAM12 in two murine PA cell lines mainly expressing estrogen receptor alpha (ER‐α, 7), AtT‐20 and TtT/GF. Since to date, no human PA cells are available, these cell lines might serve as a model for human studies.

## Materials and methods

### Reagents

Selective estrogen receptor modulators (bazedoxifene, clomiphene, and raloxifene) were purchased from Biomol (Hamburg, Germany). They were dissolved in DMSO (Sigma, St. Louis, MO, USA). Batimastat was obtained from Tocris Bioscience (Bio‐Techne, Wiesbaden, Germany).

### Cells and cultivation

We obtained AtT‐20 cells from the American Type Culture Collection (ATCC, Wesel, Germany). TtT/GF cells were kindly provided by the former laboratory of Dr. med. habil. Nicolai Savaskan at Erlangen University, Germany. Cells were grown in phenol red‐free Dulbecco's modified Eagle's medium (DMEM; Gibco, Waltham, MA, USA) containing 10% fetal bovine serum (FBS; Gibco), 0.1 mg·mL^−1^ penicillin/streptomycin (Biochrom, Berlin, Germany), sodium pyruvate (1 mm; Biochrom), and nonessential amino acids (1×; Biochrom). Cells were cultivated under 5% CO_2_ at 37 °C.

### Viability assay

One hundred microlitre medium per well containing 5000 cells per well was placed in 96‐well plates for 24 h. Thereafter, SERMs were added at the indicated concentrations. After another 48 h, 50 µL CellTiter‐Glo^®^ (Promega, Mannheim, Germany) per well were added. The plate was then vigorously shaken for 10 min and incubated for 20 min at room temperature in the dark. A FLUOstar OPTIMA Microplate Reader (BMG Labtech, Offenburg, Germany) was employed to detect luminescence. All experiments on viability were conducted in triplicates.

### Invasion assay

Eight‐micrometre transwell inserts (Greiner Bio‐One, Frickenhausen, Germany) were used with 24‐well plates. The inserts were coated on the upper side with 50 μL Matrigel (Corning^®^ Matrigel^®^ Matrix, Basement Membrane Matrix Growth Factor Reduced, Corning Incorporated, Corning, NY, USA). After 1 h at 37 °C under 5% CO_2_, the Matrigel was solid. The transwell insert was then turned upside down, and 25 000 TtT/GF cells per well resp. 50 000 AtT‐20 cells per well in 50 μL medium containing 0.5% FBS were seeded on the other side. After 4 h allowed for adherence, the transwell insert was turned upside up again. To build an FBS gradient, 250 μL medium containing 10% FBS was applied on top of each Matrigel layer, while 750 μL medium containing 0.5% FBS was placed in each of the 24 wells. After 24 h allowed for invasion, cells were fixed with 4% paraformaldehyde (Sigma), treated with 0.3 % octoxynol‐9 (Triton™ X‐100 buffer; Sigma), and stained with 4′,6‐diamidin‐2‐phenylindole (DAPI; Sigma). Cells were counted in five randomly chosen viewing fields [respectively 10 randomly chosen viewing fields with the small interfering ribonucleic acid (siRNA) experiments]. The percentage of cells that had invaded into the Matrigel was determined.

### Quantitative real time‐polymerase chain reaction (qPCR)

Cells were lysated in 1 mL Qiazol (Qiagen, Hilden, Germany). After resuspension, 200 µL chloroform was added. After mixing and incubation at room temperature for 3 min, cells were centrifuged (15 600x g, 15 min, 4 °C). The upper aqueous phase was transferred into a new 1.5‐mL tube and gently mixed with 500 µL isopropanol. After 10 min at room temperature, tubes were centrifuged again (15 600x g, 15 min, 4 °C). The supernatant was removed, and the pellet was washed with 1 mL 75% ethanol followed by centrifugation and careful removal of the supernatant. The sediment was dissolved in RNAse free water. RNA quantification was performed using a NanoPhotometer NP80 (Implen, Munich, Germany). cDNA was obtained using the ‘RNA to cDNA Ecodry™ Premix Kit’ (Takara Bio Europe, Saint‐Germain‐en‐Laye, France) according to the manufacturer's protocol. Quantitative polymerase chain reaction (qPCR) analyses were performed in triplicates using the ‘Precision FAST MasterMix with ROX’ (Primer Design, Southampton, UK). QuantiTect primers for the detection of RNA encoding mouse MMP‐1, MMP‐9, MMP‐14, ADAM8, ADAM12, and Basigin were obtained from Qiagen. We used XS13 as housekeeping gene with primers (fwd‐TGGGCAAGAACACCATGATG, rev‐AGTTTCTCCAGCTGGGTTG) purchased from Microsynth (Balgach, Switzerland). A StepOnePlus™ qPCR system (Thermo Fisher Scientific, Waltham, MA, USA) was employed. Results were normalized to blank controls using the $2^{‐\Delta \Delta C_{t}}$ method to estimate relative quantities for each gene.

### Antibodies, protein extraction, and western blot

Rabbit polyclonal anti‐MMP‐14 and anti‐glial fibrillary acidic protein antibodies were purchased from Abcam (Cambridge, UK). Rabbit polyclonal anti‐ADAM12 antibody was obtained from Proteintech (Rosemont, IL, USA). Anti‐GAPDH (Abcam) and anti‐β‐tubulin (Novus Biologicals, Littleton, CO, USA) served as loading controls, horseradish peroxidase‐conjugated donkey anti‐rabbit immunoglobulin G from Abcam was used as secondary antibody.

Total protein was extracted from the cells (AtT20: 5 × 10^5^, TtT/GF: 2 × 10^5^) with radioimmunoprecipitation assay (RIPA) buffer [50 mm 4‐(2‐hydroxyethyl)‐1‐piperazineethanesulfonic acid (pH = 7.4, Sigma); 150 mm NaCl; 1% nonyl phenoxypolyethoxylethanol (NP‐40; Sigma); 0.5% sodium deoxycholate; 0.1% SDS (SERVA, Heidelberg, Germany); 10 mm phenanthroline; 10 mm ethylenediaminetetraacetic acid (EDTA; Sigma)] that contained phosphatase inhibitors (Pierce™ Phosphatase Inhibitor Mini Tablets; Thermo Fisher Scientific), and protease inhibitors (Pierce™ Protease Inhibitor Mini Tablets, EDTA free; Thermo Fisher Scientific). In a 6‐well plate, 200 μL RIPA buffer per well was added to the cells and incubated for 15 min on ice. Shearing of the DNA was accomplished by using a 25‐G needle to resuspend the samples, which were subsequently incubated for another 30 min on ice. The lysates were then centrifuged (14 400x g, 15 min, 4 °C), and the resulting supernatant, that is, the total cellular protein, was stored at −80 °C. For 5 min, lysates were boiled in Laemmli buffer (60 mm Tris/HCl, pH = 6.8; 2% SDS; 10% glycerol; 5% β‐mercaptoethanol; 0.01% bromophenol blue). A bicinchoninic acid assay (Thermo Fisher Scientific) was used to determine protein concentrations.

Proteins from equal amounts of lysate were separated by SDS/PAGE. Proteins were then transferred onto nitrocellulose membranes. To block any unspecific binding, membranes were immersed and gently shaken in 5% nonfat, dried milk in Tris‐buffered saline Tween‐20 [TBST; 50 mm Tris, pH = 7.5; 150 mm NaCl; 0.1% Tween‐20 (CARL ROTH, Karlsruhe, Germany)] for 1 h, after which they were incubated with primary antibodies at 4 °C overnight. After three washing steps with TBST, blots were incubated with the secondary antibody for 1 h. After another washing step, signals were detected with Western Bright Chemiluminescence Substrate Sirius (Biozym Scientific, Hessisch Oldendorf, Germany) and chemiluminescence was determined using a Chemostar Imager (Intas, Göttingen, Germany) and imagej (National Institutes of Health (NIH), Bethesda, MD, USA) for quantification. Several exposure times were compared to confirm linearity of the signals.

### siRNA transfection

FlexiTube Gene Solution siRNA for mouse ADAM12, mouse MMP‐14, and AllStars‐negative control siRNA (Qiagen) was applied in AtT‐20 and TtT/GF cells. Transfection was carried out using HiPerFect reagent (Qiagen) according to the manufacturer's protocol with 5 nm siRNA applied. The resulting silencing of proteins was confirmed by western blotting as described above. Invasion rates were established according to the invasion assay protocol given above.

### Statistics

graphpad prism version 8.0.1 (GraphPad Software, San Diego, CA, USA) was used to fit the dose–response curves using nonlinear regression, to estimate the IC_25_ and IC_50_ values, and to calculate protein band densities as assessed with imagej fiji (https://imagej.net), page visited on August 21, 2020) on a Mac OS X (Apple Inc., Cupertino, CA, USA).

For the invasion assay and the qPCR, statistical analysis was conducted and figures were created with r studio version 1.1.383 (R Studio, Boston, MA, USA, https://rstudio.com, page visited on May 21, 2020) running r version 4.0.0 ‘Arbor Day’ (The R Foundation, Vienna, Austria, https://www.r‐project.org, page visited on May 21, 2020) on a Mac OS X (Apple Inc.).

If not indicated otherwise, data are presented as mean values ± standard error of the mean (SEM). *t* served as statistical test, and *P* values of < 0.05 were considered statistically significant. To assess differences in multiple group means, ANOVA and *t* testing with a correction for type I error were applied.

## Results

**Fig. 1:**
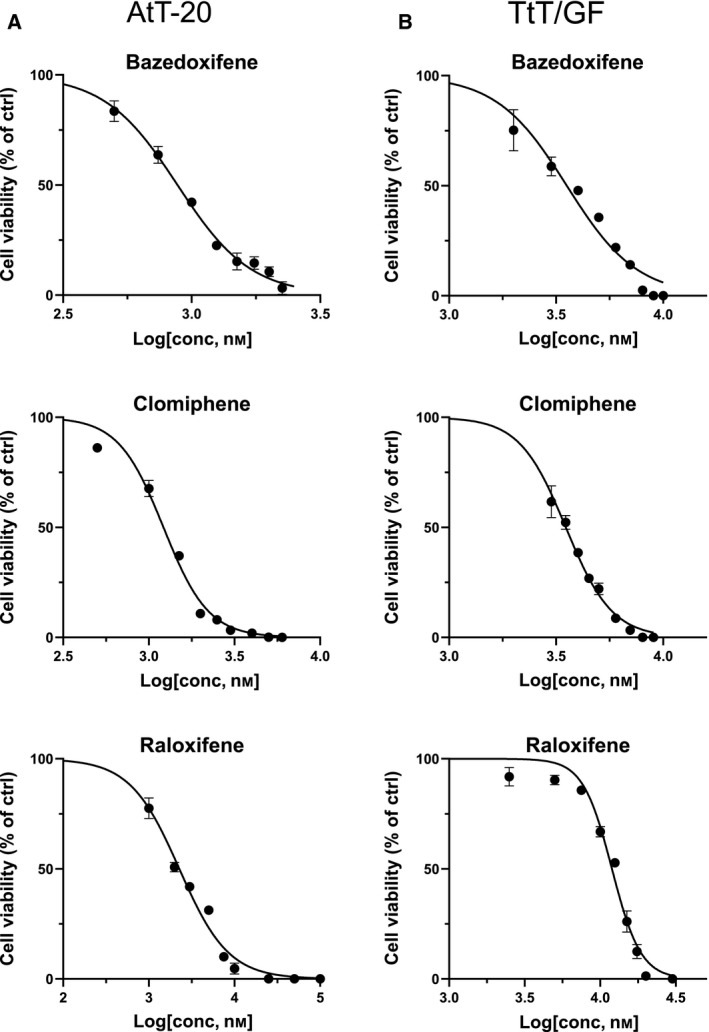
Dose–response curves for SERMs bazedoxifene, clomiphene, and raloxifene in PA cell lines AtT‐20 (A) and TtT/GF (B). Values are indicated as mean ± SEM. ctrl, control.

At first, we determined dose–response curves for the three SERMs bazedoxifene, clomiphene, and raloxifene in AtT‐20 and TtT/GF cells. According to the 50% inhibitory concentration (IC_50_) values, we found that in AtT‐20 cells, bazedoxifene exerted the most pronounced impact on viability, while in TtT/GF cells clomiphene had the highest impact on viability which was comparable to that of bazedoxifene in the same cells (Fig. 1A,B, Table 1).

**Table 1:** Summary of IC_25_ and IC_50_ values for different anti‐estrogens (in μm) for each cell line as determined by dose–response curves. Concentrations as indicated in this table were used for all other experiments in this study.

	Bazedoxifene	Bazedoxifene	Clomiphene	Clomiphene	Raloxifene	Raloxifene
	IC_25_	IC_50_	IC_25_	IC_50_	IC_25_	IC_50_
AtT‐20	0.61	0.88	0.87	1.21	1.10	2.27
TtT/GF	2.34	3.54	2.67	3.51	9.37	11.94

### SERMs decrease invasiveness in murine PA cells

**Fig. 2:**
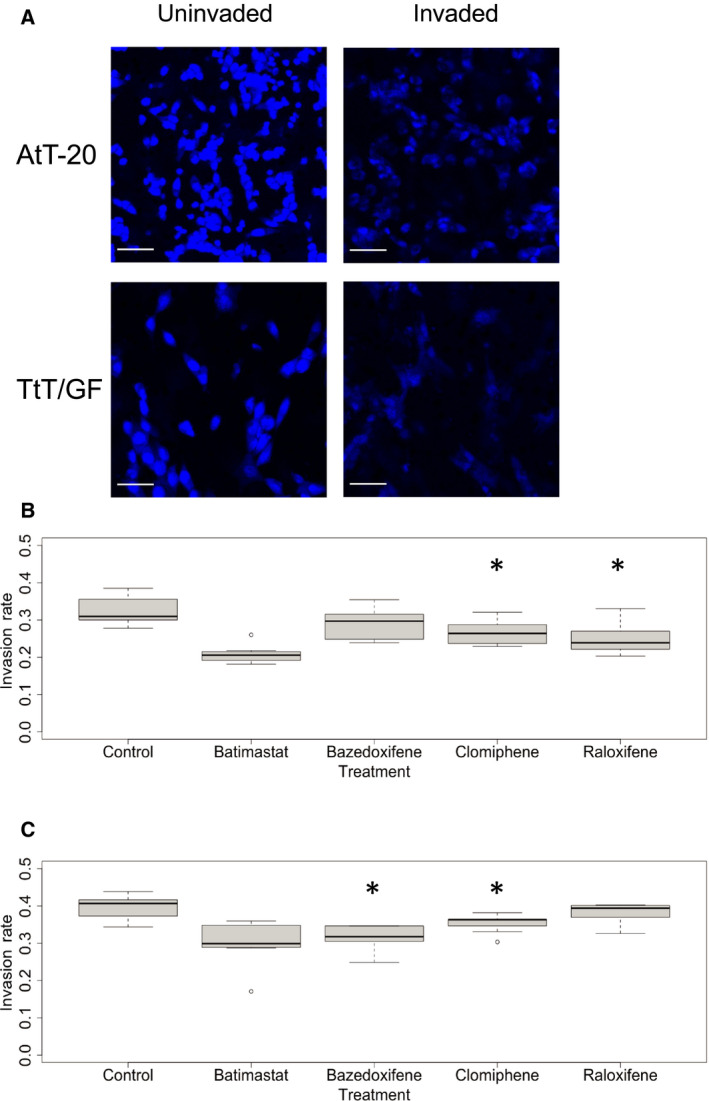
Selective estrogen receptor modulators reduce invasiveness in PA cell lines AtT‐20 (B) and TtT/GF (C). (A) Representative images of AtT20 (upper panel) and TtT/GF (lower panel) cells stained with DAPI. Note that cell lines were treated with 25% inhibitory concentrations (IC_25_) of SERMs bazedoxifene, clomiphene, and raloxifene as indicated in Table 1. Scale bar in (A), for all images, 50 μm. Batimastat, a broad‐range MMP inhibitor, served as positive control. With bazedoxifene in TtT/GF cells, the outlier (o) was excluded from the statistical analysis. Values are given as median, first quartile, and third quartile. Values are obtained from three independent experiments performed in triplicates. *t* served as statistical test. *Statistically significant difference as compared to control.

Invasion assays using Matrigel were performed with AtT‐20 and TtT/GF cells (Fig. 2A). We observed a significant impact on invasiveness after treatment of AtT‐20 cells with clomiphene (*P* = 0.027) and raloxifene (*P* = 0.009) and after treatment of TtT/GF cells with clomiphene (*P* = 0.022) and bazedoxifene (*P* = 0.002) at the respective IC_25_ (Fig. 2B,C). The effects of the three SERMs at IC_25_ were compared to control media and to the effect of batimastat, a broad‐range MMP inhibitor, which also exerted a significant effect on invasiveness in both cell lines (*P* < 0.01, Fig. 2B,C).

### SERM treatment decreases MMP‐14 and ADAM12 mRNA expression in murine PA cells

**Fig. 3:**
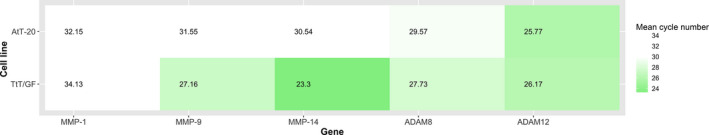
Mean cycle number values as determined by qPCR representing absolute gene expression levels of MMP‐1, MMP‐9, MMP‐14, ADAM8, and ADAM12 in untreated PA cell lines AtT‐20 and TtT/GF.

To analyze those metalloproteases affecting invasiveness of PA cells, we determined the mean qPCR cycle numbers representing absolute values of MMP‐1, MMP‐9, MMP‐14, ADAM8, and ADAM12 gene expression in untreated AtT‐20 and TtT/GF cells. Whereas MMP‐1, MMP‐9, and ADAM8 showed low expression levels, we detected high gene expression levels of MMP‐14 in TtT/GF cells and of ADAM12 in both cell lines (Fig. 3). These results prompted us to particularly investigate the potential impact of SERMs on *MMP‐14* and *ADAM12* gene expression in AtT‐20 and TtT/GF cells.

**Fig. 4:**
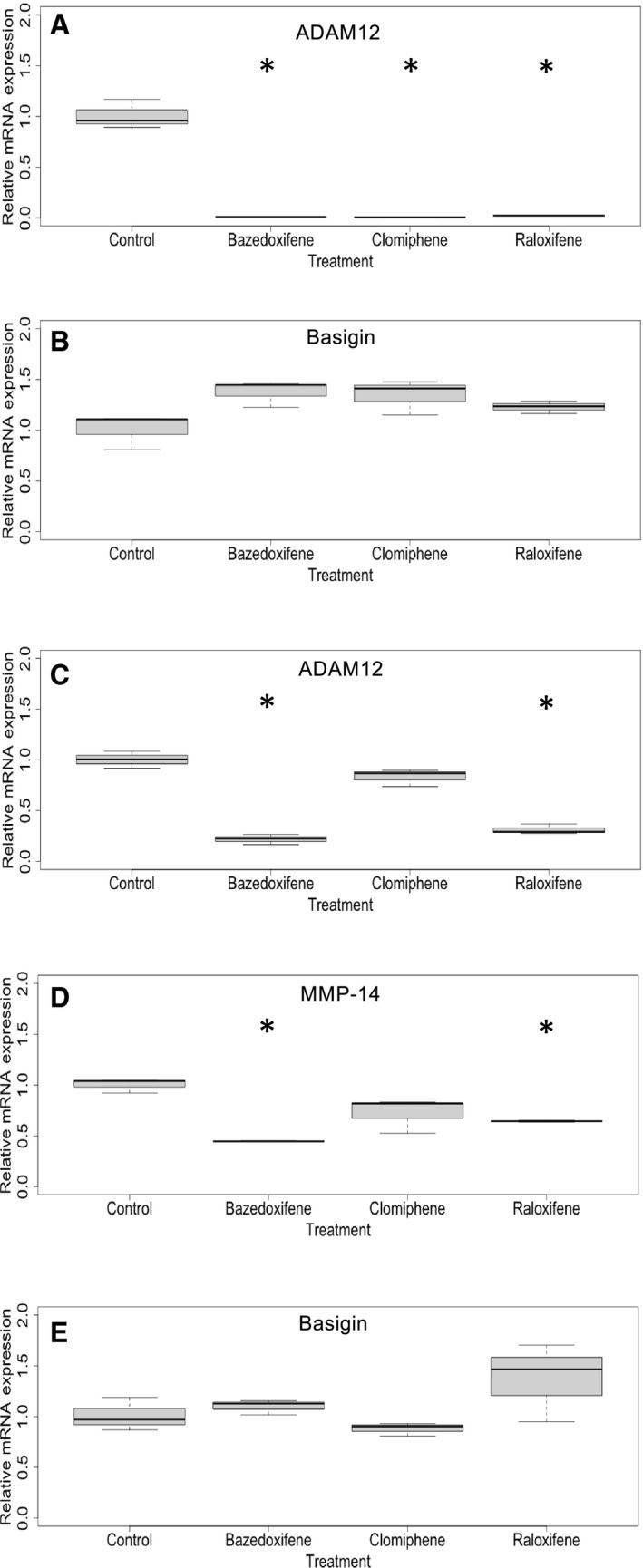
Quantitative real‐time‐polymerase chain reaction to determine mRNA levels of ADAM12 in AtT‐20 (A), of Basigin in AtT‐20 cells (B), of ADAM12 in TtT/GF cells (C), of MMP‐14 in TtT/GF cells (D), and of basigin in TtT/GF cells (E) in response to SERM treatment. For each SERM (raloxifene, bazedoxifene, and clomiphene), 50% inhibitory concentrations (IC_50_) as indicated in Table 1 were used to treat cells for 72 h. Quantitative PCR was performed in three independent experiments in triplicates. Values are given as median, first quartile, and third quartile. *t* served as statistical test. *Statistically significant difference as compared to control.

We could demonstrate a significant impact of bazedoxifene (*P* < 0.01), clomiphene (*P* < 0.01), and raloxifene (*P* < 0.01) on mRNA gene expression of ADAM12 in AtT‐20 cells. We further observed a significant impact of bazedoxifene (*P* < 0.01) and raloxifene (*P* = 0.01) on the mRNA gene expression of MMP‐14 in TtT/GF cells, as well as a significant impact of bazedoxifene (*P* < 0.001) and raloxifene (*P* < 0.001) on the mRNA gene expression of ADAM12 in TtT/GF cells, each as compared to blank controls (Fig. 4A,C,D). With or without SERMs, a relevant gene expression of MMP‐14 in AtT‐20 cells was not detected (Fig. 3, data after SERM treatment not shown). In either cell line, the gene expression levels of ADAM‐related Basigin were not significantly downregulated by SERM treatment (Fig. 4B,E).

### Gene silencing of ADAM12 and MMP‐14 decreases invasiveness of murine PA cells

**Fig. 5:**
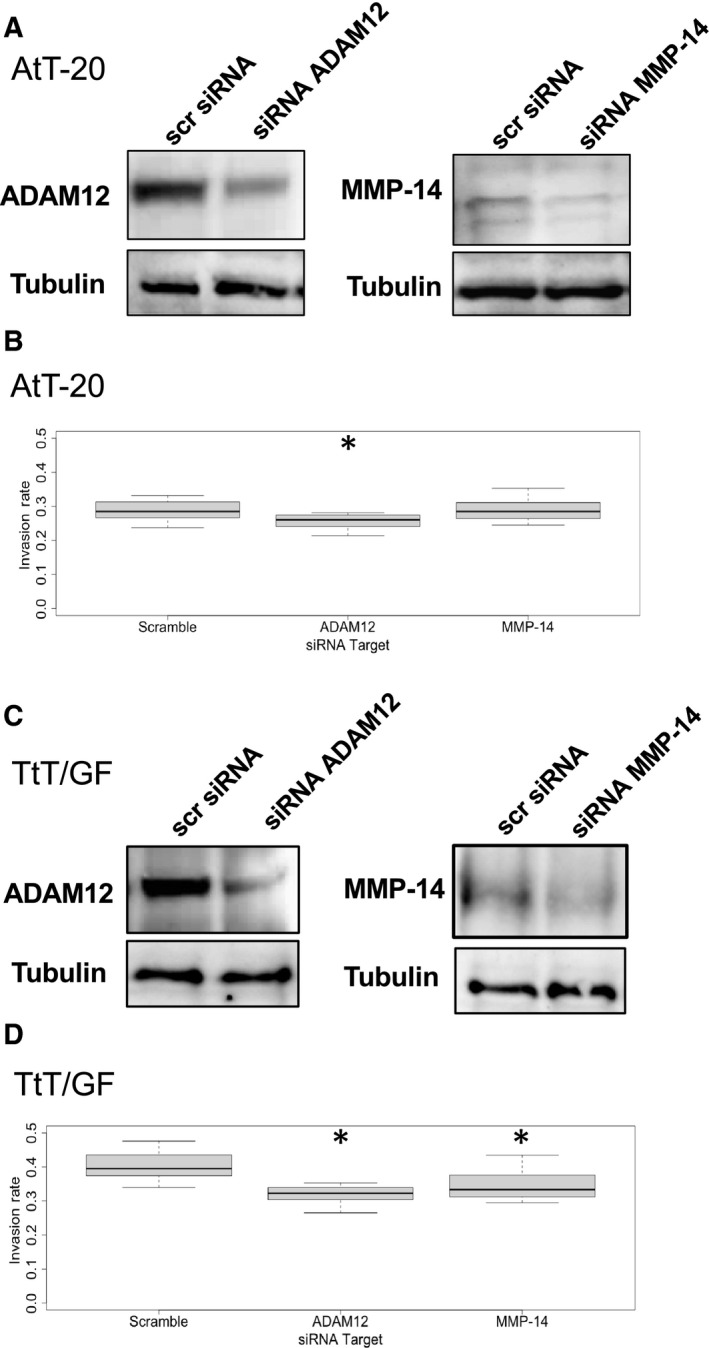
Transfection of AtT‐20 (A, B) and TtT/GF (C, D) cells with siRNA silencing *ADAM12* and *MMP‐14* genes and their effect on invasion. Western blots for the respective proteins in AtT‐20 (A) and TtT/GF (C) cells; scramble (‘scr’) siRNA served as transfection and tubulin as loading controls. Invasion rates after selective silencing of *ADAM12* and *MMP‐14* genes as compared to scramble siRNA in AtT‐20 (B) and TtT/GF cells (D) were calculated. Values are obtained from three independent experiments in triplicates and are indicated as median, first quartile, and third quartile. *t* served as statistical test. *Statistically significant difference as compared to control (Scramble).

To evaluate the role of ADAM12 and MMP‐14 in PA cell invasion, we analyzed the impact of ADAM12 and MMP‐14 gene silencing on invasiveness in AtT‐20 and TtT/GF cells. Transfection of AtT‐20 and TtT/GF cells with ADAM12 and MMP‐14 siRNA resulted in a significantly reduced protein expression of ADAM12 in both cell lines (Fig. 5A,C) and of MMP‐14 in TtT/GF cells (Fig. 5C). These cells were subjected to invasion assays and showed significantly decreased invasion rates of AtT‐20 cells after *ADAM12* silencing (*P* = 0.013, Fig. 5B), of TtT/GF cells after *ADAM12* silencing (*P* < 0.001, Fig. 5D) and of TtT/GF cells after *MMP‐14* silencing (*P* = 0.011, Fig. 5D).

### SERM treatment decreases MMP‐14 and ADAM12 protein levels in murine PA cells

**Fig. 6:**
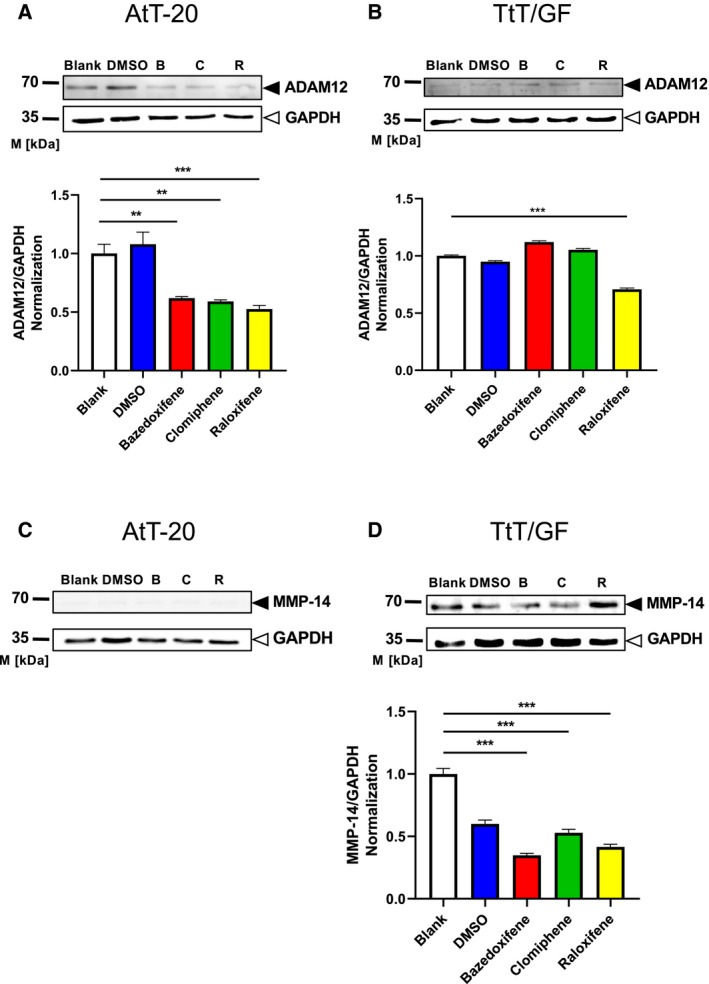
Western blot analyses of whole‐cell lysates from AtT‐20 cells (A, C) and TtT/GF cells (B, D) treated with 50% inhibitory concentrations (IC_50_, as indicated in Table 1) of SERMs bazedoxifene (B, red), clomiphene (C, green), and raloxifene (R, yellow) for three days. Protein levels of ADAM12 (A, B) and MMP‐14 (C, D) are shown from one representative experiment. Note that ADAM12 can be seen as 68‐kDa band (black arrowhead), whereas MMP‐14 is shown as a 66‐kDa band (black arrowhead) compared to solvent control (‘DMSO’). Blank served as an additional control to judge the vehicle effect. Glyceraldehyde 3‐phosphate dehydrogenase (GAPDH, 35 kDa, white arrowhead) was used as loading control. Bands were quantified by imagej analysis from three quantifications. Values are given as mean ± SEM using one‐way ANOVA. *t* served as statistical test, with **P < 0.001 and ***P < 0.0001.

In AtT‐20 cells, treatment with bazedoxifene, clomiphene, and raloxifene at IC_50_ each obviously decreased ADAM12 protein expression (Fig. 6A) as compared to DMSO and blank controls. In this cell line, an impact of the three drugs on MMP‐14 protein expression was not found according to the western blot (Fig. 6C) which, in AtT‐20 cells, hardly detected any MMP‐14 protein at all—in accordance with the qPCR results (Fig. 3).

In TtT/GF cells, treatment with bazedoxifene, clomiphene, and raloxifene at IC_50_ each obviously decreased MMP‐14 protein expression as compared to the blank control (Fig. 6D). In the same cells, treatment with bazedoxifene and raloxifene at IC_50_ also obviously decreased MMP‐14 protein expression as compared to the DMSO control, whereas treatment with clomiphene at IC_50_ exerted only a moderate effect as compared to the DMSO control. A significant impact on ADAM12 protein expression in TtT/GF cells was only found for raloxifene according to the western blot (Fig. 6B).

## Discussion

The impact of SERMs on viability and invasion in rodent PA cells and the correlation of MMP‐14 and ADAM12 expression with invasiveness in human PAs have been demonstrated previously [4, 5, 6, 7, 13]. However, this is to our knowledge the first study that connects SERMs with reduced expression levels of MMP‐14 and ADAM12, and reduced invasiveness in PA cell lines. Our findings demonstrate the potential of these drugs as inhibitors of invasion in PA cells which could become highly relevant for potential clinical applications.

A significant impact on invasion was observed after treatment of AtT‐20 cells with clomiphene and raloxifene and after treatment of TtT/GF cells with bazedoxifene and clomiphene. However, all the tested drugs moderated the invasive potential in both cell lines at least to some extent at IC_25_. These observations are somewhat different from those reported in [7], which may be explained by differences in incubation times and cell passages between studies.

ADAM12 and MMP‐14 have been found to be related to cavernous sinus invasion in human PA tissue samples [13] as well as to growth in other tumors, for example, breast cancer [14]. We found the gene expression levels of ADAM12 in AtT‐20 and TtT/GF cells and of MMP‐14 in TtT/GF cells to be significantly downregulated after treatment with bazedoxifene, clomiphene, and raloxifene. Furthermore, we found that bazedoxifene and raloxifene decreased protein expression of MMP‐14 in TtT/GF cells, and all three drugs decreased protein expression of ADAM12 in AtT‐20 cells whereas only raloxifene significantly dowregulated protein expression of ADAM12 in TtT/GF cells.

There are several limitations associated with this study. The absence of MMP‐14 gene expression in AtT‐20 cells as opposed to TtT/GF cells and the difference in ADAM12 protein expression in AtT‐20 cells as opposed to TtT/GF cells indicate that there are distinct mechanisms of invasion involved in each of the cell lines, deserving future elucidation, including investigation of the potential impact of SERMs on other invasion‐related proteases.

Another limitation is that it remains difficult to extrapolate from findings in rodent PA cells *in vitro* to potential applications in humans, since several challenges have to be overcome: In different PA cell lines from the same species, the same treatment may lead to distinct responses, as we have observed here. One may expect such differences to be similar or even more pronounced in other species. Furthermore, cultivation of human PA cells is still a major obstacle. Last but not least, apart from intravenously administered clomiphene, the concentrations of the drugs tested in this study are much higher than plasma concentrations usually achievable in humans [15, 16, 17] (Table S1).

We find it nonetheless very encouraging to see that in murine PA cells, SERMs downregulate two proteases that apparently play key roles in cavernous sinus invasion in human PAs [13].

## Conclusion

Selective estrogen receptor modulators decrease invasiveness of murine PA cell lines AtT‐20 and TtT/GF and regulate expression levels of MMP‐14 and ADAM12, two important invasion‐related proteins in murine PA cells as well as in human PA tissue samples. Our findings support the view that SERMs might become a potential salvage therapy for aggressive PAs.

## Conflict of interest

The authors declare no conflict of interest.

## Author contributions

ZZ conducted experiments and statistical analysis; JWB supervised the study and drafted the manuscript; JB conducted experiments and read the manuscript; TL supervised the study and read the manuscript; CN supervised the study, provided laboratory facilities and staff, and read the manuscript; BV conceived the project, supervised the study, conducted statistical analysis, and drafted the manuscript.

## Supporting information


**Table S1.** Molecular characteristics of the three selective estrogen receptor modulators (SERMs) that were used to treat AtT‐20 and TtT/GF cells in our study ^a^as retrieved from http://www.drugbank.ca on May 21, 2020; ^b^see reference number 15; ^c^see reference number 16; ^d^see reference number 17; for the respective inhibitory concentration (IC) values, please refer to table 1.

## Data Availability

All data will be available from the corresponding author upon reasonable request.
