# Supplementary material for: Selective estrogen receptor modulators decrease invasiveness in pituitary adenoma cell lines AtT‐20 and TtT/GF by affecting expression of MMP‐14 and ADAM12
Source: FEBS Open Bio. 2020 Oct 26;10(11):2489–98. doi: 10.1002/2211-5463.12999 (PMC7609764; doi:10.1002/2211-5463.12999)
Supplement: Supplementary file 1 — Table S1. Molecular characteristics of the three selective estrogen receptor modulators (SERMs) that were used to treat AtT‐20 and TtT/GF cells in our study aas retrieved from http://www.drugbank.ca on May 21, 2020; bsee reference number 15; csee reference number 16; dsee reference number 17; for the respective inhibitory concentration (IC) values, please refer to table 1. [file FEB4-10-2489-s001.docx]

|  | Molecular weight^a^  [g / mol] | Human peak plasma concentration (HPPC)  [ng / mL] | HPPC  [µmol / L] | IC_25_ [µmol/L]  in AtT-20 cells | IC_25_ [µmol/L]  in TtT/GF cells |
| --- | --- | --- | --- | --- | --- |
| Bazedoxifene | 470.61 | 3.43^b^ | 0.0073 | 0.6147 | 2.340 |
| Clomiphene | 405.96 | 489^c^ | 1.2046 | 0.8736 | 2.669 |
| Raloxifene | 473.58 | 0.7^d^ | 0.0014 | 0.9991 | 9.371 |

Supplementary Table S1: Molecular characteristics of the three selective estrogen receptor modulators (SERMs) that were used to treat AtT-20 and TtT/GF cells in our study ^a^as retrieved from http://www.drugbank.ca on May 21, 2020; ^b^see reference number 15; ^c^see reference number 16; ^d^see reference number 17; for the respective inhibitory concentration (IC) values, please refer to table 1
